# Tetrandrine sensitizes nasopharyngeal carcinoma cells to irradiation by inducing autophagy and inhibiting MEK/ERK pathway

**DOI:** 10.1002/cam4.3356

**Published:** 2020-08-11

**Authors:** Jun Wang, Zhouzhou Yao, Xiaoping Lai, Hongwei Bao, Yue Li, Shuaixiang Li, Lihong Chang, Gehua Zhang

**Affiliations:** ^1^ Department of Otolaryngology‐Head & Neck Surgery The Third Affiliated Hospital of Sun Yat‐sen University Guangzhou China

**Keywords:** autophagy, MEK/ERK pathway, nasopharyngeal carcinoma, radiosensitization, tetrandrine

## Abstract

Radioresistance was the main reason for local recurrence and metastasis of nasopharyngeal carcinoma. Tetrandrine is reported as an antitumor drug via inducing cell cycle arrest and apoptosis. In this study, the radiosensitization effects of maximum noncytotoxic doses of tetrandrine in nasopharyngeal carcinoma were analyzed both in vitro and *in vivo*, using MTT assay, western blot, TUNEL, and HE staining. It was found that the maximum dose of tetrandrine inhibited the phosphorylation of ERK and MEK induced by irradiation, and significantly enhanced irradiation‐induced cell growth inhibition in nasopharyngeal carcinoma cells CNE1, CNE2, and C666‐1. The ERK activator and overexpression of ERK reversed the radiosensitization effect of tetrandrine. About 50 mg/kg of tetrandrine which was used as the maximum noncytotoxic dose of tetrandrine in *vivo*, enhanced the radiosensitivity of the xenograft tumor and increased the apoptosis rate of the xenograft tumor cells caused by irradiation, while did not raise the side effect of the treatment. Moreover, tetrandrine increased autophagy in nasopharyngeal carcinoma cells. These results suggested that the maximum noncytotoxic dose of tetrandrine sensitized nasopharyngeal carcinoma to irradiation by inhibiting MEK/ERK pathway and inducing autophagy.

## Graphical abstract

We found that the maximum non‐cytotoxic dose of tetrandrine had radiosensitization effect on nasopharyngeal carcinoma cells via inhibiton MEK/ERK pathway and induced autophagy. 50mg/kg tetrandrine enhanced the radiosensitivity of the xenograft tumor in vivo, while did not raise the side effect of the treatment. It was suggested that tetrandrine would be a potential radiosentizer for nasopharyngeal carcinoma.

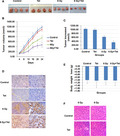

## 1 INTRODUCTION

Nasopharyngeal carcinoma is one of the common malignant tumors in South China. The age‐standardized incidence rate of nasopharyngeal carcinoma in southern China has reached 20 per 100 000, while that is more than 1 per 100 000 person in most areas of the world.^1^ Radiotherapy is the most important treatment modality for nasopharyngeal carcinoma due to the complicated anatomical structure and undifferentiated non‐ keratinizing type as the main pathological type.^2^ While the 5‐year overall survival rate can be as high as 80% for stage I and IIA patients with radiotherapy treatment alone.^3^ Up to 70% of patients with advanced (Stages III‐IV) disease at the time of diagnosis due to the atypical early clinical symptoms of nasopharyngeal carcinoma.^4^, ^5^ Concurrent chemoradiotherapy has been pivotal treatment for patients with advanced nasopharyngeal carcinoma, however, it is invariably associated with higher incidences of hematological and non‐hematological acute toxic effects compared with radiotherapy alone.^6^ Therefore, it is of great significance to find an effective and safe radiosensitizer for the treatment of nasopharyngeal carcinoma.

Tetrandrine, C_33_H_42_N_2_O_6_, is extracted from the roots of Chinese herb *Stephania tetrandra*.^7^ It was used as potential calcium channel blocker,^7^ and also was reported by its selective inhibiting of the ion channel, Eag1 channel that plays important roles in tumor proliferation, malignant transformation, invasion, metastasis and so on.^8^ It also has antihypertensive, anti‐inflammatory, and anti‐viral effects,^9^ and is currently used for rheumatoid arthritis, joint pain, and silicosis in clinic.^10^ Studies on malignant glioma, neuroblastoma, and esophageal cancer found that tetrandrine could inhibit the growth of tumor cells and could increase the sensitivity of tumor cells to radiotherapy.^11^, ^12^, ^13^ In our previous study, we found that the maximum non‐toxic doses of tetrandrine in human nasopharyngeal carcinoma cell lines CNE1 and CNE2 were 1.5 μmol/L and 1.8 μmol/L, respectively. These maximum noncytotoxic doses of tetrandrine could increase the radiosensitivity of CNE1 and CNE2, and the mechanism might be associated with the activation of CDC25C/CDK1/cyclinB1 pathway and abrogating the G2/M arrest induced by irradiation.^14^ Previous studies found that tetrandrine would regulate cell cycle and induce apoptosis or autophagy via regulating RAS mediated MAPK pathway, Akt, WNT signaling pathway, and intrinsic caspase pathway.^7^ Moreover, MAPK pathway was reported to regulate G2/M arrest in human leukemia cells^15^ and the endometrial cancer cells.^16^ As a result, in this study, we intend to investigate the roles of the maximum dose of tetrandrine in regulating MAPK pathway and autophagy and its effect on nasopharyngeal carcinoma in vivo.

## 2 MATERIALS AND METHODS

### 2.1 Cell culture and transfection

The human nasopharyngeal carcinoma cell lines CNE1 and CNE2 were obtained from Sun Yat‐sen University Cancer Center (Guangzhou, China) and had been authenticated by STR profiles on May 26th and May 29th, 2016. The human nasopharyngeal carcinoma cell line C666‐1 was obtained from Guangzhou Cellcook Biotech Co., Ltd. and had been authenticated by STR profiles on Sep 30th, 2019. Detail cell culture conditions were described as previous study reported.^14^

ERK overexpression vector (pcDNA3.1‐ERK) was constructed by Hanbio Company Limited. The empty construct pcDNA3.1 plasma was transfected as a control group. Cell transfections were conducted with Lipofectamine 3000 reagent (Invitrogen) following the manufacturer's instructions.

### 2.2 Preparation of tetrandrine

Tetrandrine purchased from Zhejiang Haizheng Pharmaceutical Company Limited (Taizhou) was dissolved in 1 mL 1 mol/L HCL, and was diluted to the final concentrations before use. The details were described in the previous study.^14^

### 2.3 Reagents and antibodies

TBHQ (tert‐Butylhydroquinone) was used as ERK activator and was purchased from MedChemExpress. Rabbit polyclonal anti‐p62 (5114s, cell signaling technology) antibody, rabbit polyclonal anti‐LC3 antibody (GeneTex, GTX127375), and rabbit polyclonal anti‐human GAPDH (10494‐1‐AP, Proteintech) were used. Rabbit monoclonal anti‐cyclin B1 (ab32053, abcam), rabbit monoclonal phospho‐CDC25C‐Ser216 (E190), rabbit polyclonal phospho‐CDK1‐Tyr15 (ab47594), and mouse monoclonal CDK1 (ab18) antibodies were purchased from Abcam (USA). Rabbit monoclonal phospho‐p38 MAPK (Thr180/Tyr182) (D3F9) antibody (4511T), rabbit monoclonal p38 MAPK (D13E1) antibody (8690T), rabbit monoclonal phospho‐SAPK/JNK (Thr183/Tyr185) antibody (81E11), and rabbit polyclonal JNK antibody (9252) were purchased from Cell Signaling Technology.

### 2.4 Western blot analysis

Total proteins were extracted from cells after different treatments and then were quantified by BCA protein assay kit (Thermo Scientific). All the western blots were repeated independently three times. The detail protocols and the antibodies used were reported as previously studies.^14^, ^17^, ^18^

### 2.5 In vivo tumor biology

Nude 5‐week‐old female BALB/c nu/nu mice (18‐22 g) were purchased from the Experimental Animal Center of Sun Yat‐sen University (Guangzhou, China). All the animals were group‐housed under specific pathogen free (SPF) conditions in Experimental Animal Center of Sun Yat‐sen University, using a laminar airflow rack, had continuous access to sterilized food and autoclaved water, with a 12 hours light/dark cycle, controlled temperature and humidity. Experiments commenced after 1 week of acclimatization.

CNE1 cells were injected subcutaneously into the right flank (3 × 10^6^ cells in 200 μL of RPMI‐1640 medium). After nearly 10 days, the xenograft volumes reached approximately 100 mm^3^. Then, the transplanted mice were randomly divided into groups (n = 5 mice each): normal saline (NS) group (0.2 mL of saline/day) and groups treated with different doses of tetrandrine (25, 50, 75, 100 mg/kg/d). All doses of tetrandrine and the NS control were administered by intragastric administration once every other day. The tumor volumes were determined by measuring length (*l*) and width (*w*) and calculating volume (*V* = 0.5 × *l* × *w* ^2^) every 4 days. The person performing tumor measurements was different from the person treating the animals so the measurements were performed in a blinded fashion. Twenty four days after irradiation, the tumors of control mice reached 1000 mm^3^, mice of all groups were sacrificed and the tumors were removed and submerged in 4% paraformaldehyde for further analysis. The mouse whose tumor size was far different from others was excluded in the final analysis. The dose of tetrandrine which significantly inhibited the tumor growth in the tetrandrine treated mice compared with the control mice but did not induce the significant body weight loss was defined as the maximum noncytotoxic dose in vivo. In order to determine the radiosensitive capacity of this maximum noncytotoxic dose of tetrandrine, and further explore its toxicity, we did the second xenograft experiment. CNE1 cells were injected in the Nude 5‐week‐old female BALB/c nu/nu mice for xenografts as described above. When the xenograft volumes reached approximately 100 mm^3^, the transplanted mice were randomly divided into 4 groups (NC control, maximum noncytotoxic dose of tetrandrine treatment, 6Gy group, and 6Gy combined maximum noncytotoxic dose of tetrandrine treatment). Twenty four hours after irradiation, mice were treated by oral administration of tetrandrine of appropriate concentration or normal saline once every other day. Tumor sizes measurement was same as descried above. The mice were euthanized by cervical dislocation 24 days after irradiation. The animal use protocol was reviewed and approved by the Animal Ethical and Welfare Committee (AEWC) of Sun Yat‐sen University. The approval no. is IACUC‐DB‐17‐0104.

### 2.6 Hematoxylin‐eosin staining

Tissues for hematoxylin‐eosin staining were fixed with 10% neutral buffered formalin, processed and trimmed, embedded in paraffin, sectioned to a thickness of approximately 5 μm. Then the sections were stained with eosin solution for 3 minutes and followed by dehydration with graded alcohol and clearing in c.

### 2.7 TUNEL Staining

The experiment was carried out strictly according to the instructions of TUNEL kit (Millipore). Briefly, the sections were pretreated with 3% H_2_O_2_ for 15 minutes in the dark and then were digested with proteinase K (20 μg/mL in 10 mmol/L Tris/HCl, pH 7.5) at 37°C for 15 minutes. After rinsed in phosphate‐buffered saline (PBS) for three times, the sections were immerged in TUNEL reaction mixture in a humidified chamber at 37°C for 1 hour, followed by the incubation with peroxidase streptavidin conjugate for 30 minutes at 37°C. The slides were further visualized using a diaminobenzidine (DAB) kit (Bioworld, China). A negative control was set up using the Label Solution instead of the TUNEL reaction mixture. The images of each section were captured under a light microscope (Carl Zeiss), and Image‐Pro Plus 6.0 was used to calculate the number of apoptotic cells. Ten slices were randomly selected and the apoptotic index was calculated.

### 2.8 Statistical analysis

All data were described as the Mean ± SE. Student's *t* tests were performed using SPSS 16.0. *P* < .05 was considered statistically significant.

## 3 RESULTS

### 3.1 The maximum noncytotoxic dose of tetrandrine enhanced irradiation‐induced cell growth inhibition in CNE1, CNE2, and C666‐1

**FIGURE 1:**
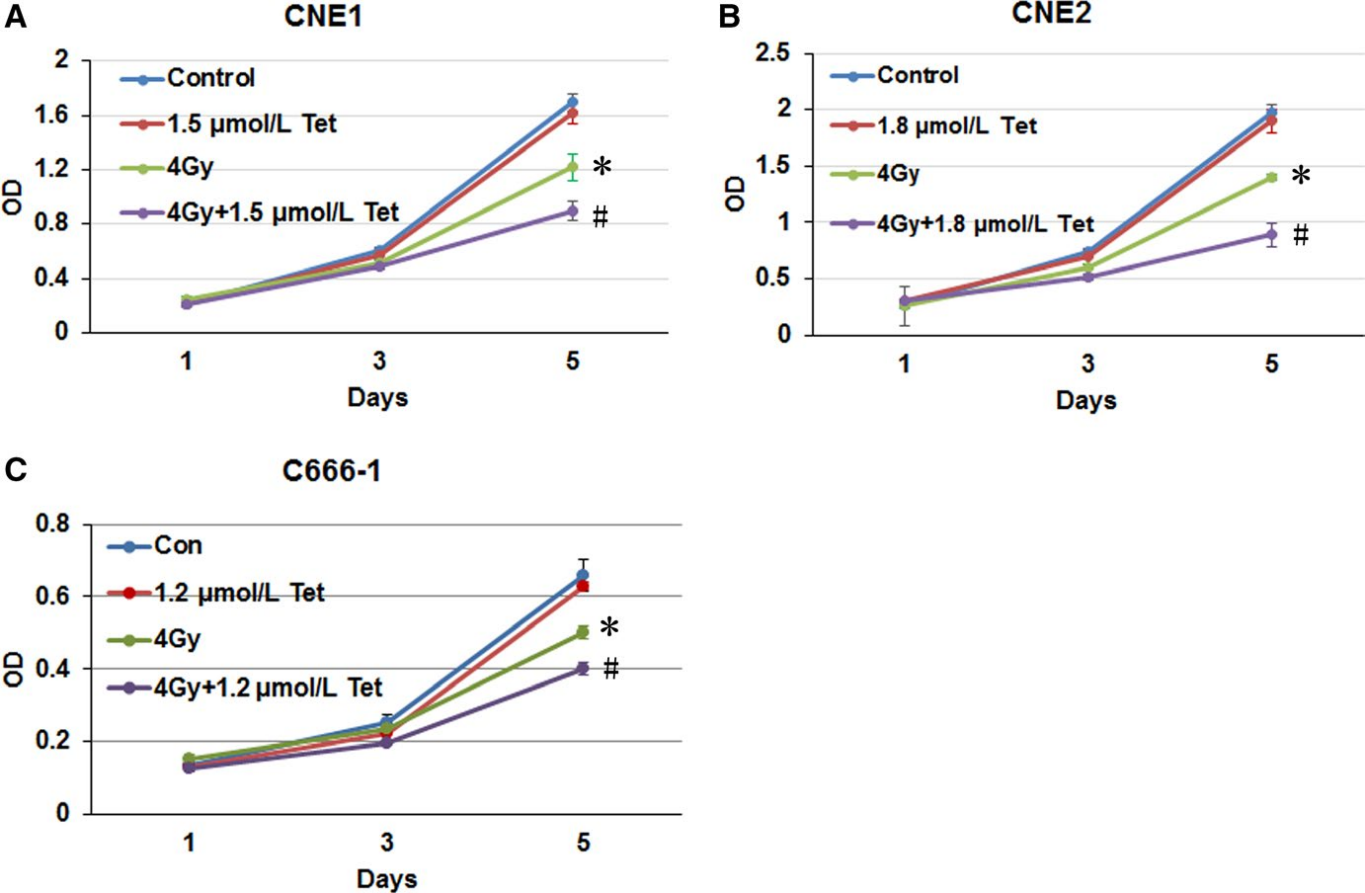
The maximum noncytotoxic does of tetrandrine enhanced the cell growth inhibition induced by irradiation. (A), (B), and (C) were the cell growth curves of CNE1, CNE2, and C666‐1 cell lines treated with tetrandrine, 4Gy irradiation alone or combination. The data were shown as the mean ± SE. * *P* < .05 vs control, #*P* < .05 vs 4Gy irradiation

The maximum noncytotoxic doses of tetrandrine were 1.5 μmol/L for CNE1 and 1.8 μmol/L for CNE2, as our previous study reported, the maximum noncytotoxic dose of tetrandrine for C666‐1 was 1.2 μmol/L (Figure S1A,B). Tetrandrine would enhance the inhibition of irradiation on the growth of CNE1, CNE2, and C666‐1 cells (Figure 1A‐C).

### 3.2 The maximum noncytotoxic dose of tetrandrine inhibited phosphorylation of MEK and ERK

**FIGURE 2:**
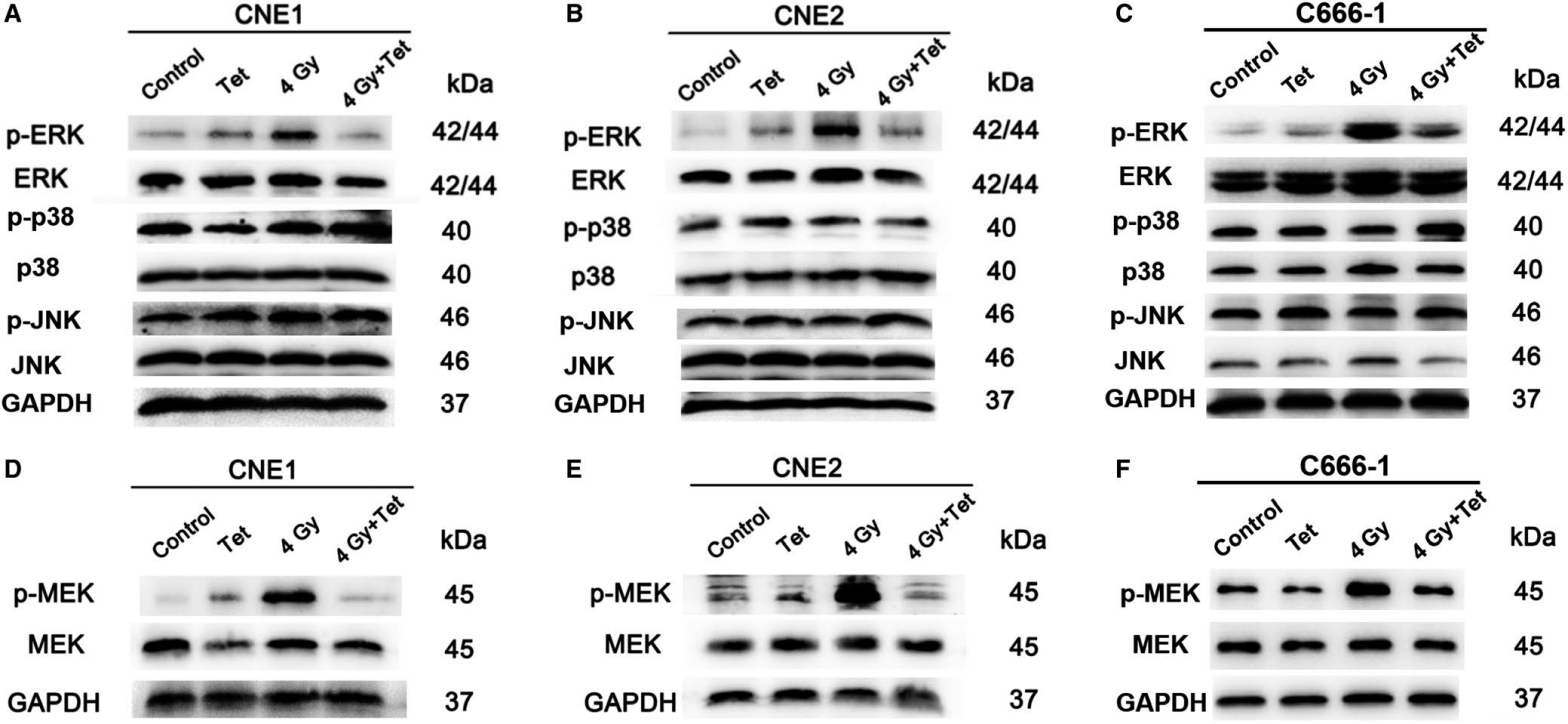
The expressions of MAPKs in nasopharyngeal carcinoma cell lines with different treatments (A)‐(C) were the expressions of MAPK family members in CNE1 cell line, CNE2 cell line and C666‐1 cell line, respectively. (D)‐(F) were the expression of p‐MEK and MEK in CNE1 cell line, CNE2 cell line and C666‐1 cell line, respectively (Control: control group, Tet: treatment of the maximum noncytotoxic doses of tetrandrine, 4Gy: treatment of 4Gy irradiation, 4Gy + Tet: combined treatment of 4Gy irradiation and the maximum noncytotoxic doses of tetrandrine)

The expression of family members of MAPK was detected in each cell line by western blot. In CNE1 cells, as shown in Figure 2A, the expression of p‐ERK showed no significant difference between the control group and tetrandrine group (*P* > .05), while that was increased after irradiation treatment (*P* < .05), and decreased after the combination treatment of irradiation and tetrandrine (*P* < .05). However, there was no significant difference in ERK, p‐p38, p38, p‐JNK, and JNK among the groups. The same trend was observed in CNE2 cells and C666‐1 (Figure 2B,C).

To investigate the upstream factor of ERK, we detected the expression of p‐MEK, an upstream kinase. As shown in Figure 2D‐F, in CNE1, CNE2, and C666‐1 cells, the combined treatment of irradiation and the maximum noncytotoxic dose of tetrandrine inhibited the increase of p‐MEK expression which was caused by irradiation (*P* < .05).

### 3.3 ERK activation and overexpression reversed the sensitization effect of the maximum noncytotoxic dose of tetrandrine

**FIGURE 3:**
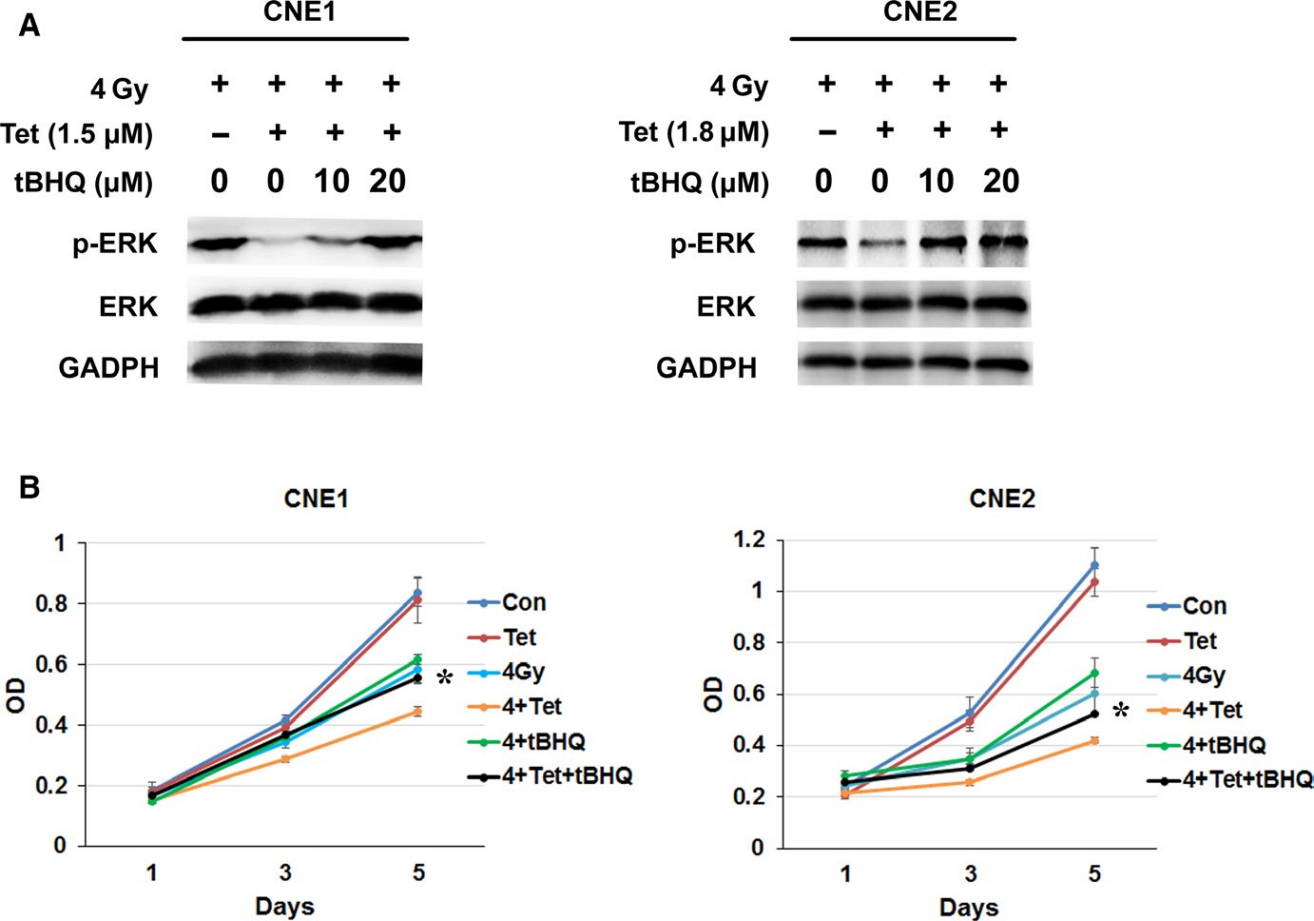
ERK activator stimulation weakened the radiosensitization effects of the maximum noncytotoxic dose of tetrandrine. (A) CNE1 and CNE2 cell lines were treated with ERK activator, tBHQ or tetrandrine treatment after 4Gy irradiation for 2h. After 24hours treatments, total proteins were extracted for detecting the expressions of phosphorylated ERK, ERK by western blot. (B) The growth curve of CNE1 and CNE2 cells after different treatments. (4Gy: treatment of 4Gy irradiation, Tet: maximum noncytotoxic doses of tetrandrine treatment, 4Gy + Tet: combined treatment of 4Gy irradiation and the maximum noncytotoxic doses of tetrandrine, 4Gy + tBHQ: combined treatment of 4Gy irradiation and followed 20 μM tBHQ treatment; 4Gy + Tet+tBHQ: combined treatment of 4Gy irradiation and followed maximum noncytotoxic doses of tetrandrine and 20 μM tBHQ treatment). *P < .05 was 4Gy + Tet+tBHQ vs 4Gy + Tet

**FIGURE 4:**
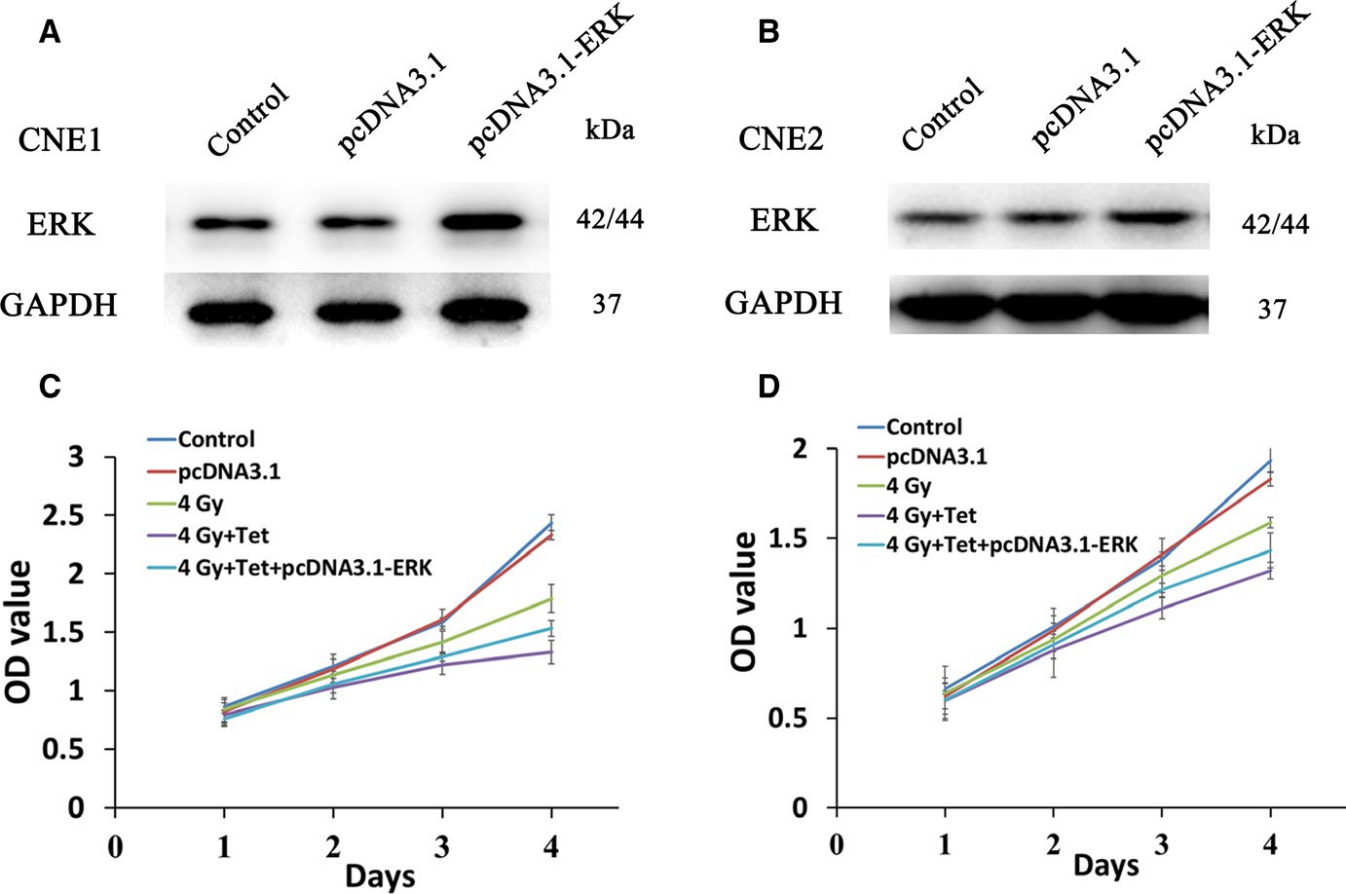
The ERK overexpression weakened the radiosensitization effects of the maximum noncytotoxic doses of tetrandrine. (A) The expression of ERK in CNE1 cells after transfection of pcDNA3.1 or pcDNA3.1‐ERK. (B) The expression of ERK in CNE2 cells after transfection of pcDNA3.1 or pcDNA3.1‐ERK. (C) The growth curve of CNE1 cells after different treatments. (D) The growth curve of CNE2 cells after different treatments. (Control: control group, pcDNA3.1: transfection of pcDNA3.1, 4Gy: treatment of 4Gy irradiation, 4Gy + Tet: combined treatment of 4Gy irradiation and the maximum noncytotoxic doses of tetrandrine, 4Gy + Tet+pcDNA3.1‐ERK: combined treatment of 4Gy irradiation and the maximum noncytotoxic doses of tetrandrine after transfection of pcDNA3.1‐ERK)

To further verify the role of ERK in the radiosensitization mechanism of the maximum noncytotoxic dose of tetrandrine, both ERK activator and ERK overexpression vector were used. MTT assay was used to detect the radiosensitization effect of tetrandrine after stimulation by ERK activator tBHQ or transfection of ERK overexpression plasmid (pcDNA3.1‐ERK). The cells were transfected with ERK overexpression plasmid for 72 hours and then treated with irradiation. The tBHQ or tetrandrine was added 2 hours before 4Gy irradiation. As Figure 3A demonstrated, 1.5 μmol/L tetrandrine significantly inhibited the phosphorylation of ERK in CNE1, 20μM tBHQ would significantly increase the expression of p‐ERK inhibited by tetrandrine. Similarly, both 10 and 20 μmol/L tBHQ would increase the expression of p‐ERK inhibited by 1.8 μmol/L tetrandrine in CNE2. 20 μmol/L tBHQ treatment would significantly reverse the enhanced role of the maximum noncytotoxic dose of tetrandrine on cell growth inhibition caused by irradiation in CNE1 and CNE2 (*P* < .05) (Figure 3B). As shown in Figure 4A,B, the expressions of ERK in CNE1 and CNE2 were upregulated after the transfection of pcDNA3.1‐ERK (*P* < .05). The results of MTT assay in CNE1 were shown in Figure 4C. Compared with irradiation group, combined treatment of irradiation and tetrandrine with or without ERK overexpression both enhanced the inhibition of cell growth caused by irradiation (*P* < .05). However, ERK overexpression attenuated the inhibition of combined treatment partially (*P* < .05). The same trend was observed in CNE2 cells (Figure 4D).

### 3.4 The maximum noncytotoxic dose of tetrandrine induced autophagy

**FIGURE 5:**
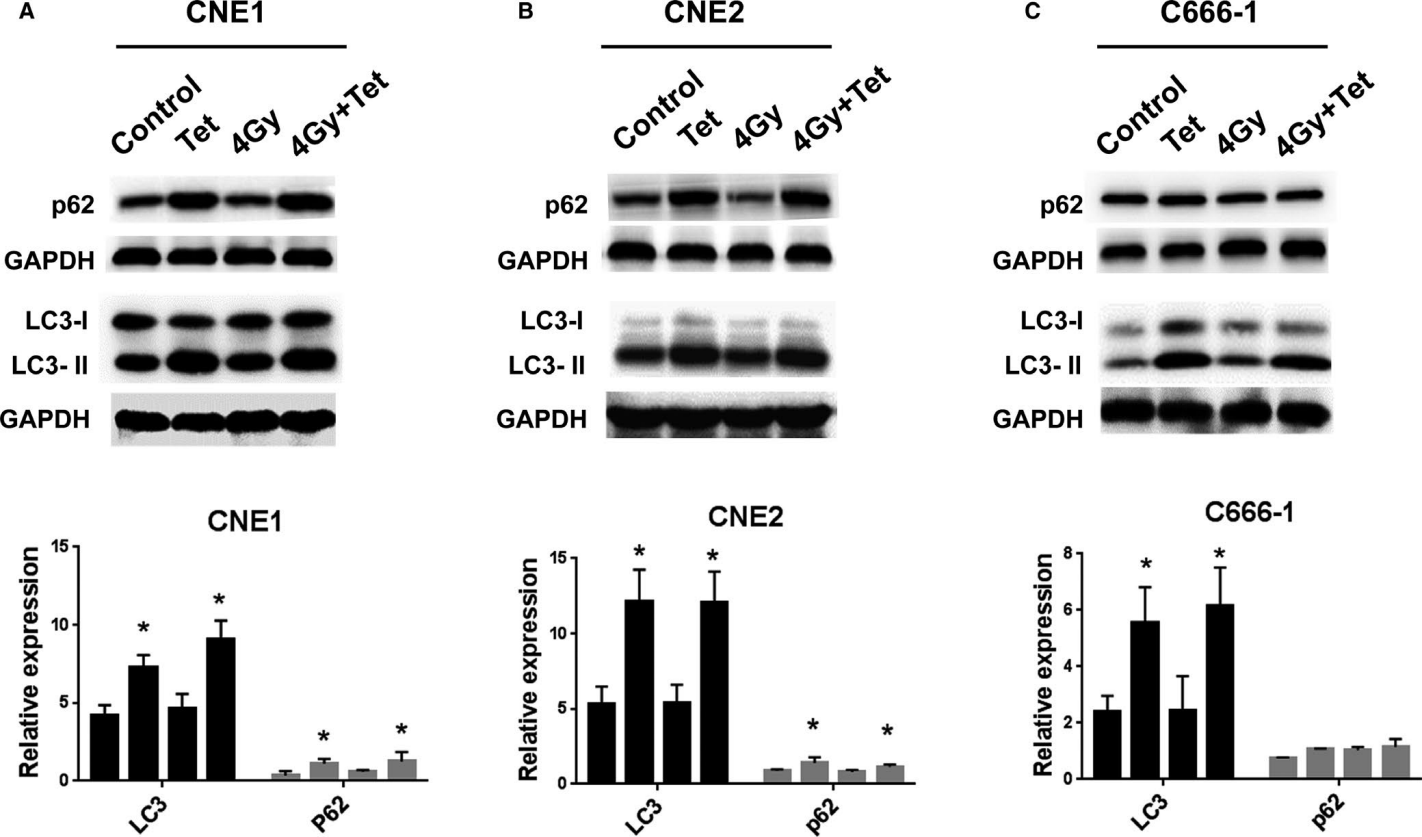
The maximum noncytotoxic dose of tetrandrine induced autophagy in NPC cell lines. (A) The western blot detection of LC3 and p62 expressions in CNE1 cell lines after different treatments. Relative expressions of LC3 and p62 in CNE1 cell lines with different treatments was conducted using GraphPad 7.0. Significant increased LC3 expressions were found in CNE1 cells treated with the maximum noncytotoxic dose of tetrandrine alone or combined with 4Gy irradiation when compared with control or 4Gy irradiation alone, using t test. (B) and (C) were the western blot detection and Relative expressions comparisons of LC3 and p62 in CNE2 and C6661‐1, respectively. The values represented the mean ± SD. *P < .05, **P < .01

It was reported that the inhibition of MEK1/2 leads to activation of the LKB1 → AMPK→ULK1 signaling axis, to elicit autophagy.^19^ Moreover, our previous study pointed out that induced autophagy could enhance the radiosensitivity of NPC cells. So, in this study, we detected the autophagy‐related proteins, p62 and LC3 expressions after the maximum noncytotoxic dose of tetrandrine treatment. It was demonstrated that the maximum noncytotoxic dose of tetrandrine treatment alone or combination with irradiation induced the upregulation of both p62 and LC3 in CNE1 (Figure 5A), CNE2 (Figure 5B) and C666‐1 (Figure 5C). The Akt‐mTOR pathway which would impact on the autophagy was also detected. It was demonstrated that the maximum noncytotoxic dose of tetrandrine induced the activation of Akt and mTOR (Figure S2).

### 3.5 The maximum noncytotoxic dose of tetrandrine inhibited the tumor growth without increasing side effect

In order to determine the effect of maximum noncytotoxic dose of tetrandrine on NPC radiosensitivity in vivo, the human nasopharyngeal carcinoma xenograft model was established and treated with normal saline, 25, 50, 75, and 100 mg/kg tetrandrine once every other day to found out the maximum noncytotoxic dose of tetrandrine. As shown in Figure S3A, the volume of xenograft tumor showed no significant difference among the control group, 25 mg/kg tetrandrine group and 50 mg/kg tetrandrine group, but the growth of tumor was significantly inhibited in 75 and 100 mg/kg tetrandrine group (*P* < .05). As shown in Figure S3B, the body weight of the mice in 75 and 100 mg/kg tetrandrine group was significantly lower than that of the control group, 25 mg/kg tetrandrine group and 50 mg/kg tetrandrine group (*P* < .05). These results demonstrated that 50 mg/kg tetrandrine had no obvious influence on the tumor growth and the body weight of mice. Therefore, we used 50 mg/kg as the maximum noncytotoxic dose of tetrandrine in xenograft tumor in the following researches.

**FIGURE 6:**
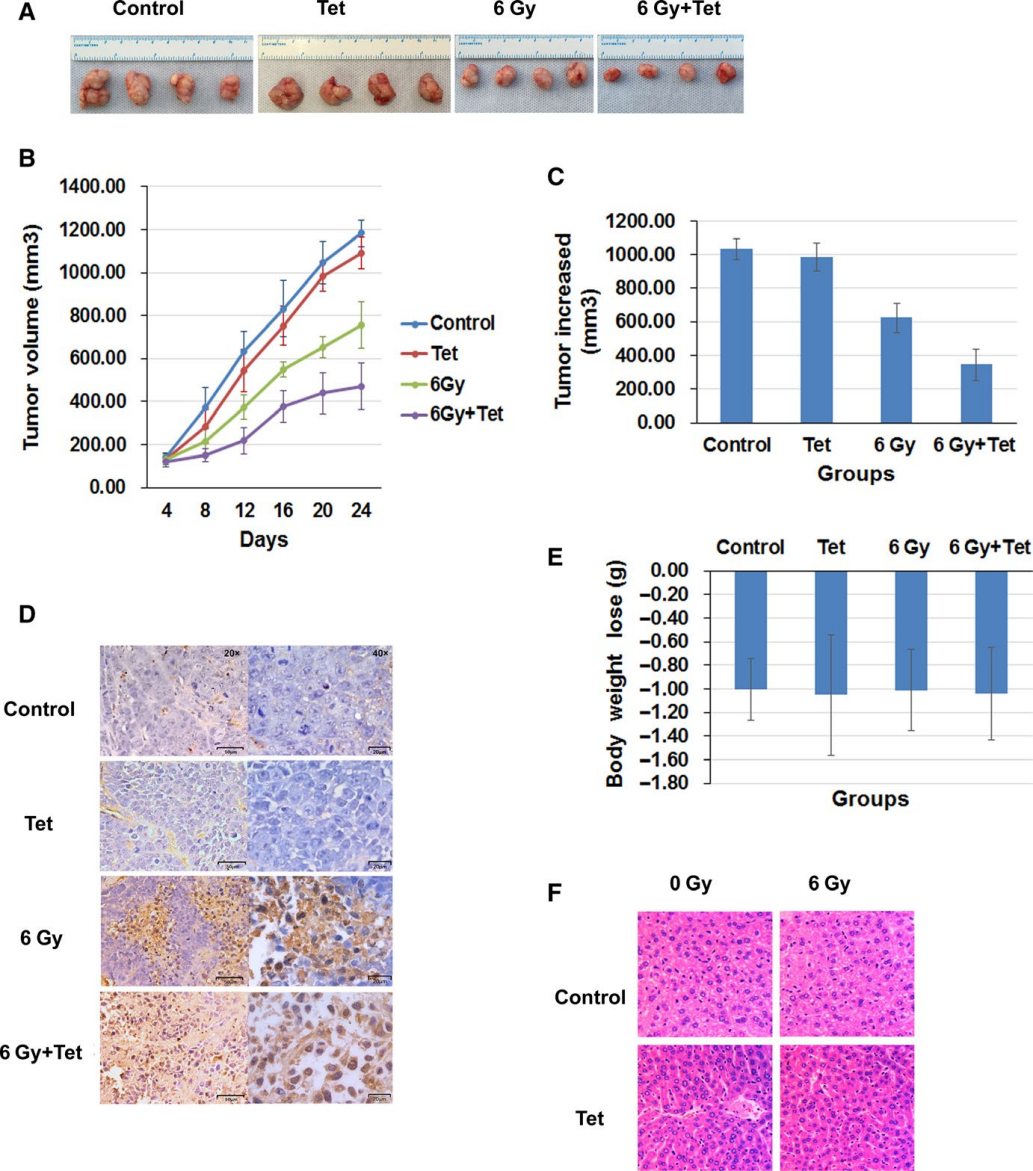
The maximum noncytotoxic dose of tetrandrine enhanced the radiosensitivity of the xenograft tumor without increasing side effect. (A) The image of xenograft tumor of mice with different treatments at 24th day after irradiation. (B) The growth curve of xenograft tumor of mice with different treatments. (C) The tumor increased volumes in mice with different treatments. (D) The TUNEL staining of tumor tissues in mice with different treatments. The objective magnification of the TUNEL staining was 20× (Left) and 40× (Right), respectively. (E) The body weight losses of xenograft tumor mice after different treatments. (F) The HE staining of the liver cells of xenograft tumor mice after different treatments. (Control: control group, Tet: treatment of the maximum noncytotoxic doses of tetrandrine, 6Gy: treatment of 6Gy irradiation, 6Gy + Tet: combined treatment of 6Gy irradiation and the maximum noncytotoxic doses of tetrandrine). The objective magnification was 40×. *P < .01 for 6Gy irradiation vs control or tetrandrine alone. # *P* < .01 for 6Gy irradiation combined with tetrandrine vs irradiation alone

When the volume of xenograft tumor reached about 100 mm^3^, the nude mice were randomly divided into 4 groups: the control group, the maximum noncytotoxic dose (50 mg/kg) of tetrandrine group, 6Gy irradiation group, combined treatment of 6Gy irradiation and the maximum noncytotoxic dose of tetrandrine group. As shown in Figure 6A,B, the tumor growth in irradiation group was inhibited, and the inhibition was more obvious in combined treatment of 6 Gy irradiation and 50 mg/kg tetrandrine (*P* < .05). The tumor increased volume in mice with 6 Gy treatment (623.53 ± 85.81 mm^3^) was significantly lower than those in control mice (1032.48 ± 63.51 mm^3^, *P* < .01) and mice with tetrandrine treatment (983.55 ± 86.68 mm^3^, *P* < .01). The tumor increased volume in mice with 6 Gy combined with tetrandrine treatment (344.94 ± 93.16 mm^3^, *P* < .01) was significantly lower than that in mice with 6 Gy irradiation alone (Figure 6C). What was more, as shown in Figure 6D, the proportion of TUNEL‐positive cells in xenografts of mice with combined treatment was higher than that in irradiation group (*P* < .05). These data revealed that 50 mg/kg tetrandrine could increase the radiosensitivity of xenografts and increase the apoptosis of xenografts caused by irradiation.

To investigate whether the treatment of irradiation combined with the maximum noncytotoxic dose of tetrandrine increased the toxicities, we examined the body weight of mice after different treatments. As shown in Figure 6E, there was no significant difference in the body weight loss of mice among different groups (*P* > .05). The HE staining of mouse livers (Figure 6F) showed that no significant cell necrosis or degeneration was detected in all groups, suggesting that tetrandrine did not increase the obvious side effects of combined treatment.

### 3.6 The maximum noncytotoxic dose of tetrandrine inhibited phosphorylation of ERK and MEK in vivo

**FIGURE 7:**
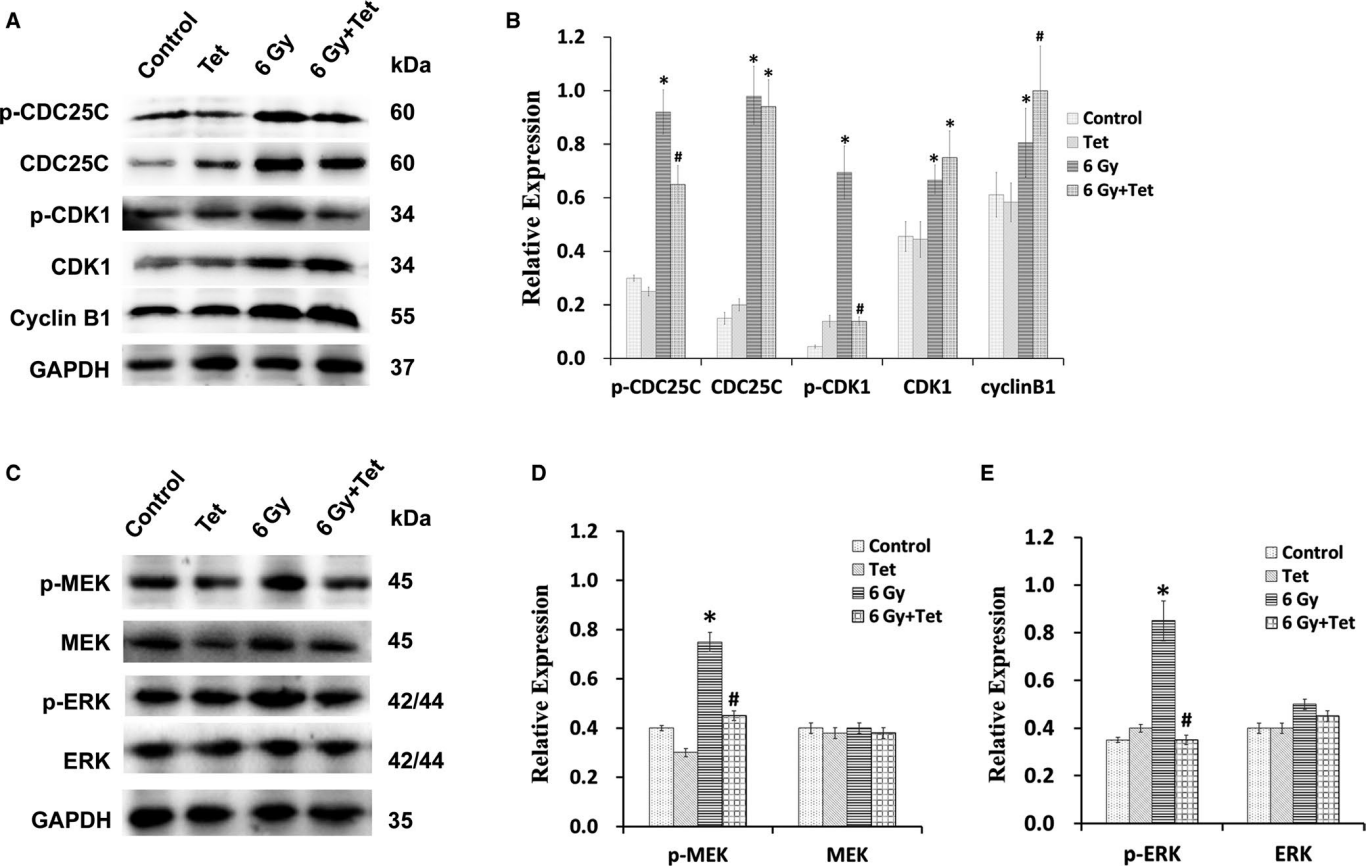
The maximum noncytotoxic dose of tetrandrine suppressed the phosphorylation of ERK and MEK and activated CDC25C/CDK1/cyclinB1 pathway in xenograft tumors. (A) The expressions of key proteins of CDC25C/CDK1/cyclinB1 pathway in xenograft tumors. (B) The relative expression of key proteins of CDC25C/CDK1/cyclinB1 pathway in xenograft tumors. (C) The western blot image of p‐MEK、MEK、p‐ERK and ERK in xenograft tumors. (D) The relative expression of key proteins of p‐MEK and MEK in xenograft tumors. (E) The relative expression of key proteins of p‐ERK and ERK in xenograft tumors. (*P < .05 vs control, #*P* < .05 vs 6Gy, Control: control group, Tet: treatment of the maximum noncytotoxic doses of tetrandrine, 6Gy: treatment of 6Gy irradiation, 6Gy + Tet: combined treatment of 6Gy irradiation and the maximum noncytotoxic doses of tetrandrine)

Our previous study pointed out that the maximum noncytotoxic dose of tetrandrine enhanced the radiosensitivity of NPC cells by regulating the CDC25C/CDK1/cyclinB1 pathway. Here, we examined the expressions of p‐CDC25C, CDC25C, p‐CDK1, CDK1, and cyclinB1 in tumor tissues from mice with different treatments by western bolt. As shown in Figure 7A,B, irradiation treatment could increase the phosphorylation of CDC25C and CDK1, which could be significantly inhibited after combined treatment of irradiation and the maximum noncytotoxic dose of tetrandrine (*P* < .05). Moreover, compared with irradiation group, the combined treatment increased the expression of cyclinB1 (*P* < .05). This tendency is consistent with those of CNE1 and CNE2 cell lines. Since MEK/ERK as the upstream factor that regulates CDC25C/CDK1/cyclinB1 cell cycle pathway as we tested in CNE1 and CNE2 cell lines, we further investigated whether the maximum noncytotoxic dose of tetrandrine could regulate the activation of MEK and ERK in vivo. The combined treatment of irradiation and 50 mg/kg tetrandrine could inhibit the upregulation of the phosphorylation of ERK and its upstream factor MEK, which was caused by irradiation (Figure 7C‐E).

## 4 DISCUSSION

Radiotherapy is the primary treatment of nasopharyngeal carcinoma, it is of great importance of enhancing the radiosensitivity of nasopharyngeal carcinoma to improve the survival rate, reduce the side effect of treatment and improve the prognosis. Our previous results suggested that the maximum noncytotoxic dose of tetrandrine could increase the radiosensitivity of human nasopharyngeal carcinoma cell lines CNE1 and CNE2 in vitro, which might be associated with the abrogation of G2/M arrest to increase the DNA damage induced by irradiation.^14^ Previous literatures pointed that tetrandrine would inhibit several cancer cells proliferation and induce cell cycle arrest by inactivating MAPK pathway or Akt pathway.^7^ In our current study, we found that the maximum noncytotoxic dose of tetrandrine would reduce the phosphorylation of the ERK, one of the main signaling of MAPK pathway, while increased the phosphorylation of Akt. It was consistent with the study that tetrandrine enhanced the radiosensitization of human glioma by attenuating ERK phosphorylation.^20^ Inconsistently, other studies pointed out that tetrandrine suppressed the phosphorylation of Akt to promote apoptosis or autophagy and induce cell cycle arrest in gastric and Triple‐Negative Breast cancer cells.^21^, ^22^ It was presumed that the maximum dose of tetrandrine would enhance the radiosensitivity of human nasopharyngeal carcinoma cells independently of Akt pathway inhibition.

MAPK signaling pathway plays an important role in regulating gene expression in eukaryotic cells and controlling fundamental cellular processes, such as growth, proliferation, differentiation and apoptosis.^23^ There are three main independent MAPK pathways composed of ERK, JNK, and p38 signaling families.^24^ Huang et al^25^ reported that ERK pathway could increase the radioresistance of glioma. Another research found that sorafenib increased the radiosensitivity of lung cancer cells by increasing ERK phosphorylation.^26^ The activation of p38 is thought to be associated with radioresistance of breast cancer.^27^ In contrast, a study on various tumor cell lines, such as lung cancer, kidney cancer, and colon cancer, concluded that p38 had no influence on cell viability after irradiation.^28^ What's more, it was reported that inhibitors of ERK could promote G2/M arrest in human leukemia cells.^15^ Whereas in the endometrial cancer cells, ERK phosphorylation induced by procyanidins could induce G2/M arrest.^16^ In our previous study, we found that the treatment with the maximum noncytotoxic dose of tetrandrine inhibited the upregulation of p‐ERK caused by irradiation. However, the phosphorylation levels of p38 and JNK did not change significantly after different treatments. It is suggested that the radiosensitization effect of the maximum noncytotoxic dose of tetrandrine on CNE1 and CNE2 was related to its inhibition of ERK phosphorylation, but not p38 and JNK.

Recent studies pointed out that the inhibition of MEK/ERK signaling would induce autophagy.^19^ In this study, we found that the maximum noncytotoxic dose of tetrandrine would induce p62 abundance or LC3 accumulation by western blots analysis. It was similar with the study on NSCLC cell lines that another Chinese medicine, curcumin, enhanced the gefitinib sensitivity through inducing dramatic and autophagy‐mediated apoptosis and downregulated MEK/ERK pathway.^29^ The link between MEK1/2 inhibition and the autophagy induction was that the MEK/ERK inhibition lead to the activation of the ULK1 → AMPK→LKB1 axis to promote autophagy.^19^ It was indicated that maximum noncytotoxic dose of tetrandrine would enhance NPC radiosensitivity by promoting autophagy via MEK/ERK inhibition. Besides, as an inhibitor of ULK, mTOR prevents ULK1 activation by phosphorylating ULK1 Ser 757 and disrupting the interaction between ULK1 and AMPK.^30^ Our previous research indicated that activating autophagy by mTOR pathway inhibition enhanced NPC cells radiosensitivity. While, in this study, we found that the maximum noncytotoxic of tetrandrine would activate mTOR but not inhibit the mTOR pathway. It was indicated that the maximum dose of tetrandrine increased autophagy and help for radiosensitivity, independent of mTOR pathway inhibition.

Maximum noncytotoxic dose of tetrandrine enhanced the radiosensitivity in NPC cell lines via MEK/ERK inhibition. Research on brain gliomas showed that oral tetrandrine (150 mg/kg/day) could inhibit tumor growth,^31^ and tetrandrine (150 mg/kg/day) intraperitoneal injection for 5 days could reduce the lung metastases of colon cancer by 40%.^32^ Zhang et al^33^ found that tetrandrine could increase the growth inhibition caused by cisplatin in an ovarian cancer xenograft tumor. However, there is limited data on tetrandrine's effect on radiosensitivity of nasopharyngeal carcinoma in vivo. Furthermore, previous studies *in vivo* did not limit the dose of tetrandrine, which may increase the side effects of treatment when combined with radiation therapy. In our research, we investigated the maximum noncytotoxic dose of tetrandrine in human nasopharyngeal carcinoma xenograft tumor and found that 50 mg/kg tetrandrine alone had no effect on the tumor growth and body weight of mice. Therefore, we used 50 mg/kg tetrandrine as the maximum noncytotoxic dose in vivo experiments. We found that combined treatment of 50 mg/kg tetrandrine and irradiation could increase the inhibition of the tumor growth in vivo, promote the apoptosis of xenograft tumor cells, and had no significant influence on body weight and liver cells of mice, suggesting that 50 mg/kg tetrandrine could increase the radiosensitivity of xenograft tumor, without increasing side effect. Then we detected the expression of the key proteins in the xenograft tumor, and found that the combined treatment of irradiation and the maximum noncytotoxic dose of tetrandrine could also inhibit the upregulation of p‐CDC25C, p‐CDK1 and p‐ERK, and p‐MEK caused by irradiation, increase the expression of cyclinB1, which is consistent with that in vitro. These results let us propose that MEK/ERK pathway and autophagy are key targets in maximum noncytotoxic dose of tetrandrine‐induced irradiation sensitization. And more attention of detail mechanism of MEK/ERK inhibition by tetrandrine and the combination therapy of tetrandrine and autophagy inductors should be deserved.

## 5 CONCLUSION

The maximum noncytotoxic dose of tetrandrine had radiosensitization effect on nasopharyngeal carcinoma cells. It would inhibit the phosphorylation of ERK and MEK induced by irradiation and induce autophagy. About 50 mg/kg of tetrandrine enhanced the radiosensitivity of the xenograft tumor in vivo, while did not raise the side effect of the treatment. It was suggested that tetrandrine would be a potential radiosentizer for nasopharyngeal carcinoma.

## CONFLICT OF INTEREST

The authors state that there are no personal or institutional conflicts of interest.

## AUTHOR CONTRIBUTION

Gehua Zhang: experimental design and supervision. Jun Wang drafted manuscript and carried out most western blot analysis and data analysis. Zhouzhou Yao helped for the western blot, MTT analysis and the revising the manuscript. Lihong Chang carried out the experimental design, the design and interpretation data, and some animal experiments. Xiaoping Lai, Hongwei Bao, Yue Li and Shuaixiang Li helped for the revising the manuscript, irradiation, ERK overexpression plasmid infection and drafted partially manuscript. All authors read and approved the final manuscript.

## Supporting information

Fig S1

Fig S2

Fig S3

## Data Availability

The datasets used and analyzed during the current study are available from the corresponding author on reasonable request.
