# Corrigendum

**DOI:** 10.1002/cam4.4173

**Published:** 2021-08-16

In the article by Wang et al,^1^ the authors would like to add two additional funding sources under funding information:

The Science and Technology Foundation of Guangdong Province (2017A020215073, 2014020212619)
