# Supplementary figures and images for: Tetrandrine sensitizes nasopharyngeal carcinoma cells to irradiation by inducing autophagy and inhibiting MEK/ERK pathway
Source: Cancer Med. 2020 Aug 11;9(19):7268–78. doi: 10.1002/cam4.3356 (PMC7541130; doi:10.1002/cam4.3356)

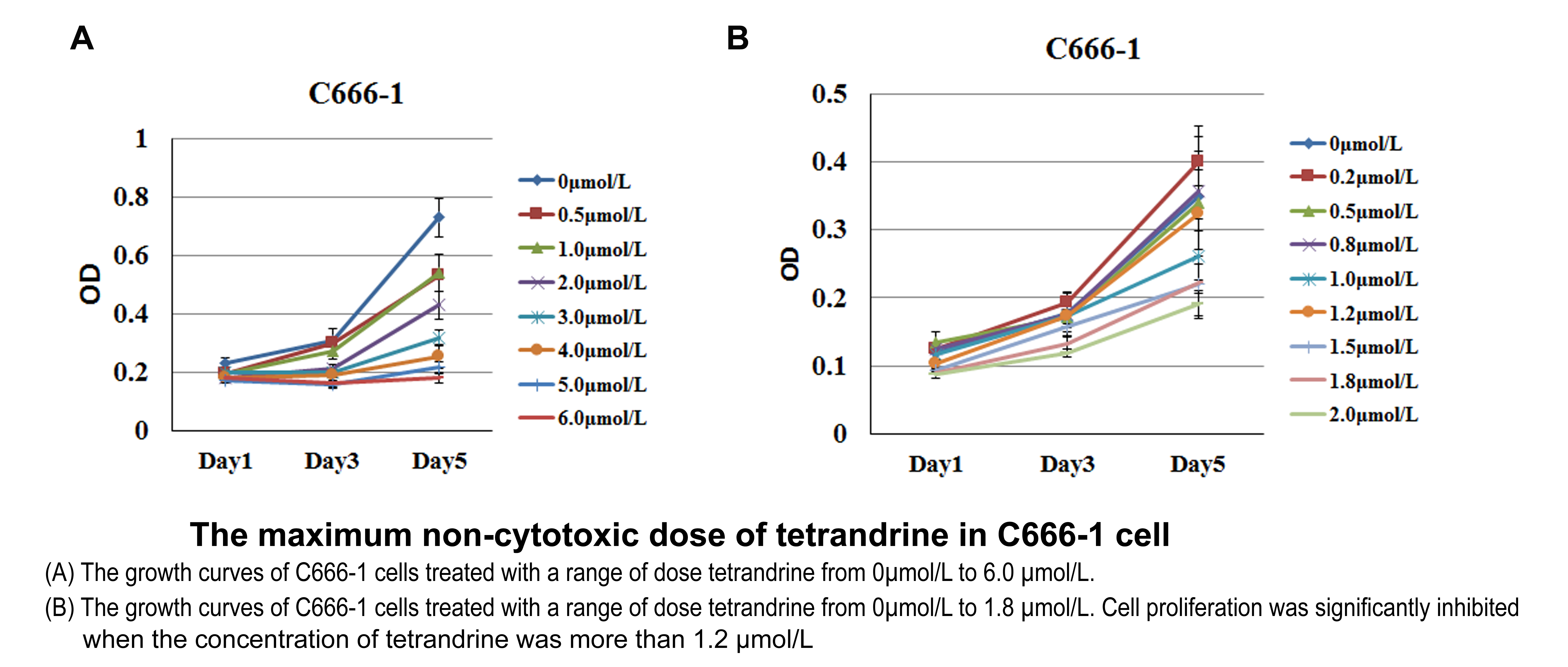

Supplement: Supplementary file 1 — Fig S1 [file CAM4-9-7268-s001.tif]

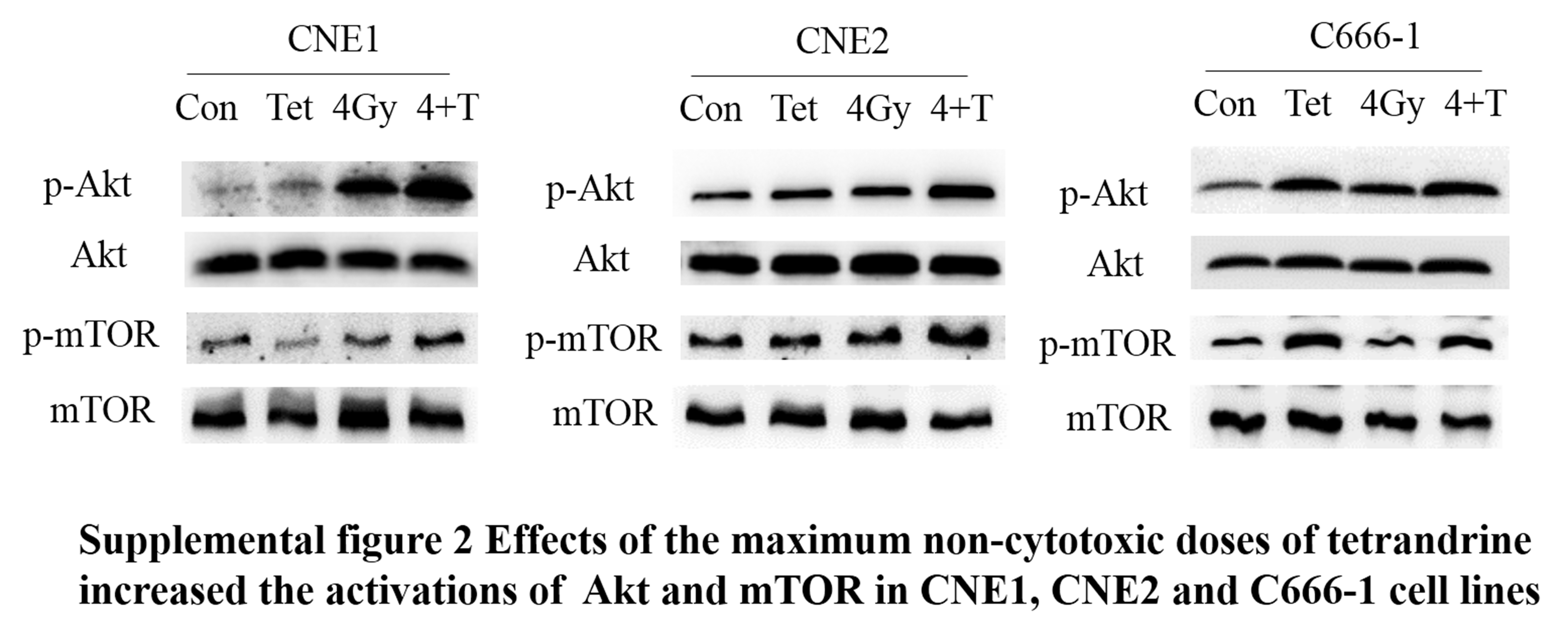

Supplement: Supplementary file 2 — Fig S2 [file CAM4-9-7268-s002.tiff]

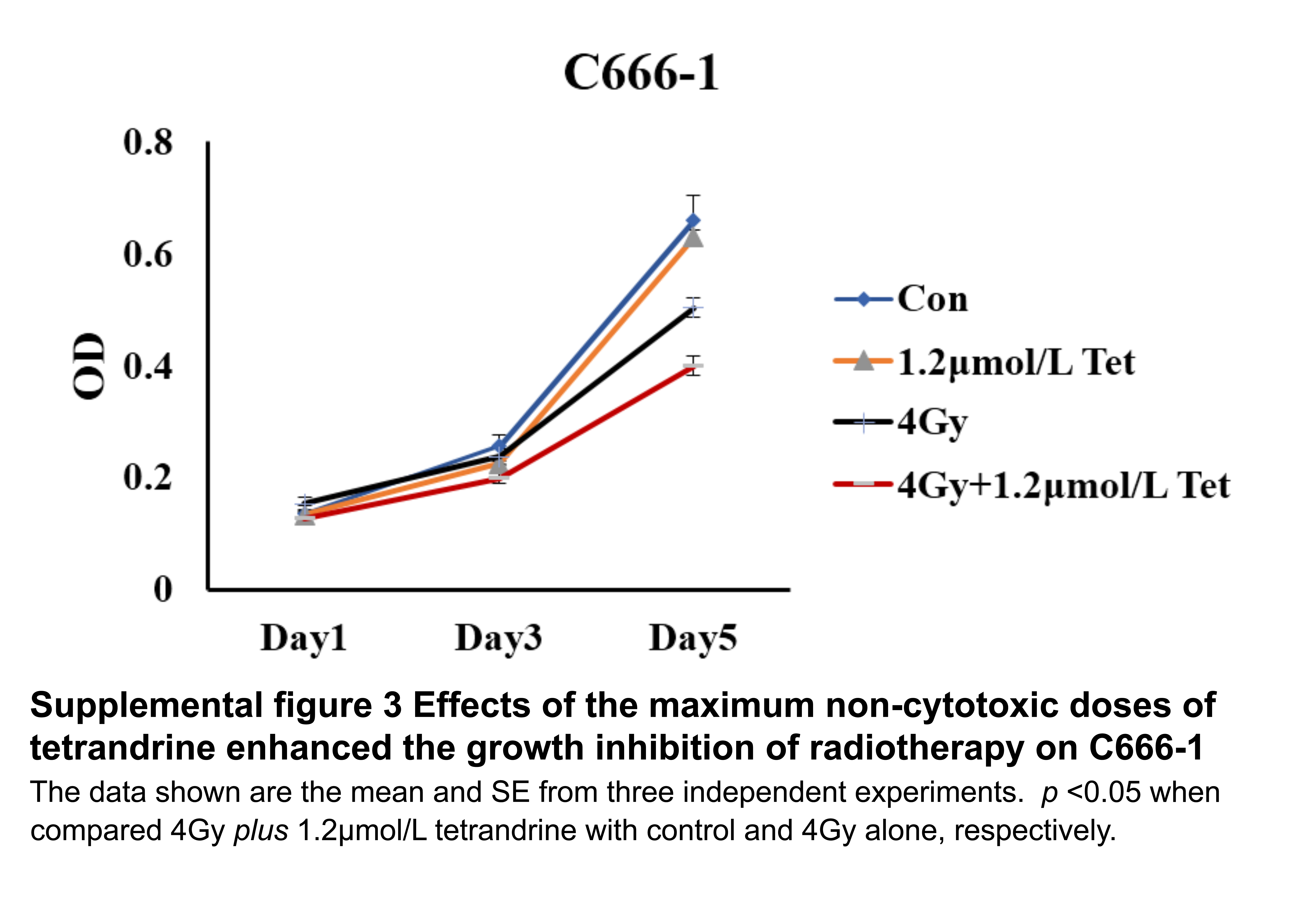

Supplement: Supplementary file 3 — Fig S3 [file CAM4-9-7268-s003.tif]
